# The Inhibitory Effects of Recombinant Hespintor Combined with Sorafenib on Transplanted Human Hepatoma in Nude Mice, and Transcriptional Regulation of Hespintor Based on RNA-Seq

**DOI:** 10.7150/jca.50500

**Published:** 2021-01-01

**Authors:** Yong-Zhi Lun, Jie Sun, Ben Liu, Wen Dong, Ling-Hong Pan, Jian Lin, Jing-Xia Zhang

**Affiliations:** Key Laboratory of Medical Microecology, Fujian Province University, School of Pharmacy and Medical Technology, Putian University, Putian 351100, China.

**Keywords:** Hespintor, Serpins, Advanced hepatocellular carcinoma, RNA-Seq, Differentially expressed genes, Reactive oxygen species

## Abstract

**Objective:** As targeted drugs, exogenous serpins could be introduced to patients to restore body balance. This study aimed to observe further the inhibitory effects of recombinant Hespintor (a Kazal-type serpin) combined with Sorafenib on transplanted human hepatoma tumors in nude mice specimens and to explore the possible transcriptional regulation by Hespintor.

**Methods:** A model of human hepatoma tumors transplanted in nude mice was established, and the medication was administrated to observe the growth of the tumors. Four weeks after the drug administration, the tumors were removed to evaluate the inhibition effects of Hespintor on in-situ tumor growth and liver metastasis. The expression levels of MMP2, MMP9, Bax, Bcl-2, and caspase-3 in the tumor organizations were detected with Western blot. The target genes of the Hespintor were screened based on tissue RNA-Seq, and the regulatory network was constructed.

**Results:** It was found that the recombinant Hespintor displayed a significant antitumor effect on the subcutaneous growth of MHCC97-H cells. Moreover, the therapeutic effects of the combination therapy were significantly better than those of single therapy. 10 target genes with significantly different expression by Hespintoron tumor tissue were identified. Finally, a visual regulatory networkwas constructed for target mRNA-pathway.

**Conclusions:** The antitumor effect of Hespintor combined with Sorafenib in treating the subcutaneously implanted hepatocellular carcinoma tumors in nude mice was significant. The possible transcriptional regulation by Hespintor involved multiple signaling pathways, and it was not just the antitumor effect of uPA via its extracellular inhibitions.

## Introduction

Human serine proteinase inhibitors (serpins) are active regulators involving many physiological processes, including blood coagulation, inflammatory responses, cell migration, fibrinolysis, complement activation, extracellular matrix reconstruction, and hormone transport and apoptosis. At present, serpins are known to be found in animals, plants, archaea, bacteria, and viruses. It has been determined that approximately 1,500 members belong to at least 20 gene families 1. Among those members, Kazal serpins are considered to be one of the more conservative families and are generally composed of one or more repeated conservative Kazal domains. In tumor cells, serine protease can induce its activation through matrix metalloproteinases (MMPs). Then, the activated MMPs hydrolyze the tumor extracellular matrix, while the serpins can indirectly inhibit the activities of the MMPs through directly inhibiting the activities of the serine protease. In this way, the migration and invasion abilities of tumor cells are controlled 2.

As early as 2005, this research group used suppression subtractive hybridization (SSH) technology to study HBV RT/DNA polymerase trans-regulated target genes. A new gene with unknown functions was screened from the HepG2 cell line of hepatoblastoma. It was confirmed by RT-PCR verification and bioinformatics that the gene belonged to a new Kazal serpin, which was subsequently named "Hespintor" and submitted to NCBI GenBank (Accession No. DQ438947) in March of 2006. Then, in December of 2006, the reference sequence of the gene (Accession No. NM_001040129) was determined, and by June of 2018, it was officially updated and renamed as a serine peptidase inhibitor Kazal type 13 (spink13). Hespintor cDNA is 285 nucleotides in total length, encoding 94 amino acids (aa), and includes three domains: N-terminal 1-23 aa is a signal peptide; the 35-94 aa contains a typical Kazal domain, and the 24-34 aa between the signal peptide and Kazal domain forms a connection region^3^. The previous research results confirmed that the Hespintor recombinant protein could significantly inhibit the proliferation, migration, and invasion of hepatocellular carcinoma cells in-vitro. Besides, it was found to display good inhibitory effects on the subcutaneous growth of the transplanted tumors of hepatocellular carcinoma cells in nude mice, and its molecular mechanism has been preliminarily elucidated 4, 5. Therefore, based on the finding above, this study focused on observing the inhibitory effects of the recombinant Hespintor when combined with Sorafenib on transplanted tumors of the highly invasive human hepatoma cell line MHCC97-H in nude mice. Based on tissue RNA-Seq, Hespintor target genes were screened, and a regulatory network was constructed by a network module partition method.

## Materials and methods

### Ethics statement

The animal experiments in this study were carried out following the British Animal (Scientific Procedures) Act of 1986 and its relevant criterion and were approved in advance by the Institutional Animal Care and Use Committee of Putian University. In total, 44 SPF grade nude mice specimens were acquired from Chongqing Ensiweier Biotechnology Co., Ltd. The specimens were all 6 to 8 weeks old, male, and weighed approximately 22 g (±2 to 3 g). All animals were housed in an animal facility at 25 °C, with a relative humidity of 60%, in a 12 h light/12 h dark cycle, with both food and sterile tapwater ad libitum.

### Reagents, plasmids, and bacterium strain

In the present study, the rabbit anti-mouse Bcl-2, Caspase-3 monoclonal antibody (primary antibody) and rabbit anti-mouse β-actin (internal reference) were purchased from Abcam (USA); bovine serum albumin was purchased from Amersham Pharmacy (USA); trypsin (activity ≥ 250 NF U/mg) and lysozyme were supplied by Amresco (USA); PVDF membrane and EasySee Western Marker were purchased from the Bio-Rad Co. (USA); DMEM cell culture medium and fetal bovine serum were acquired from GIBCO (USA), and the Sorafenib was purchased from MedChemExpress (USA). Besides, an enhanced ECL chemiluminescence detection kit was purchased from Pierce (USA); Na-Benzoyl-DL-arginine-4-nitroanilide hydrochloride (BAPNA), nickel sulfate (NiSO_4_•12H_2_O), and sheep anti-rabbit HRP IgG (second antibody) was purchased from Sigma (USA); PageRulerPrestained Protein Ladder was obtained from Thermo (USA); and the mouse anti-human His•Tag monoclonal antibody (primary antibody) and sheep anti-mouse HRP IgG (second antibody) were supplied by Beijing Zhongshan Jinqiao Biotechnology Co. Ltd, China. The chloral hydrate utilized in this study was purchased from the Aladdin Co. of Shanghai, China, and the BCA protein concentration test kit and Western blotand IP cell lysate were purchased from Beyotime Co. of Shanghai, China. Besides, the skimmed milk powder and polyformaldehyde used in this study were obtained from SANGON (Shanghai, China). The prokaryotic expression recombinant plasmid pMAL-c5x-Hespintor and *Escherichia coli* (*E. coli*) BL21 (DE3) were preserved in our lab.

### Cell line and cell culture

In the present study, MHCC97-H, a highly invasive human hepatocarcinoma cell line, was supplied by BeinaChuanglian Co. of Beijing, China. The cells were cultured in a DMEM (1% penicillin-streptomycin) cell culture medium containing 10% (v/v) fetal bovine serum. The cells were placed in a 37 °C, 5% CO_2_ incubator for routine culturing, and passed on once every 1 to 2 d.

### Expression, purification, and activity identifications of the recombinant protein

The recombinant plasmid of pMAL-c5x-Hespintor was transformed into the competent cells of *E.coli* BL21(DE3). Then, the transformed bacterial solution was evenly coated on an LB plate containing ampicillin and cultured overnight at 37 °C. A single colony was selected from the LB broth containing ampicillin, and the colony was added to the media and cultured until OD600=0.6 to conduct an inducible expression at 0.8 mM IPTG and 37 °C for 4 h. At the same time, BL21(DE3)/pMAL-c5x-Hespintor without an inducible expression and BL21(DE3)/pMAL-c5x with BL21(DE3)/pMAL-c5x were used as the controls. The bacterial precipitation was collected by centrifugation, and the samples were heated and boiled. Then, the expression results were analyzed using a 12% SDS-PAGE.

The above-mentioned transformed bacteria solution was then used as seed for the enlarged cultivation process. When OD600 reached 0.6, an inducible expression process was conducted at 0.2 mM IPTG and 18 °C for 16 h. The bacteria were then collected and lysed with ultrasonic waves, and the supernatant was collected. The purified recombinant protein was obtained using a Ni-NTA affinity chromatography method and dissolved in PBS by buffer replacement and detected with a 12% SDS-PAGE. As detailed in Reference 4, the purified Hespintor protein solution's concentration was measured without adding the recombinant protein as a negative control. Then, the enzyme inhibition activities were successfully detected. The dosage effects on the inhibition of trypsin hydrolysis were calculated according to the following formula, and the inhibition curve was drawn:


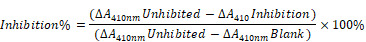


### Modeling and grouping of the experimental animals

MHCC97-H cells at the logarithmic growth stage were selected to prepare cell suspensions with 1×10^7^ cells/mL concentrations. Each of the nude mice specimens was inoculated with 200 μL cell suspensions under its right armpit. After bearing tumors for one day, the nude mice were randomly divided into four groups, with 11 specimens in each group, and treatments were administered two days later. For the recombinant Hespintor treatment group, the Hespintor solution was injected into the caudal vein at a 20 μg/kg dosage, and intragastric administrations were made with normal saline at a 2 mg/kg dosage. In the Sorafenib treatment group, normal saline was injected into the tail veins at a 20 μg/kg dosage, and intragastric administrations were made with Sorafenib solution at a 2 mg/kg dosage. For the combined administration group, the Hespintor solution was injected into the tail veins at a 20 μg/kg dosage, and intragastric administrations were made with Sorafenib solution at a 2 mg/kg dosage. For the solvent control group, normal saline was injected into the tail veins at a 20 μg/kg dosage, and intragastric administrations were made with normal saline at a 2 mg/kg dosage. The administration times of tail vein injections in the four transplanted tumor model groups were two times per week during the first 2 weeks and three times per week during weeks 3 to 4. The intragastric administrations were carried out every second day. The aforementioned experimental scheme or method has been approved by the Ethics Committee of Putian University.

### Antitumor effect of the recombinant protein *in vivo*

Following the nude mouse's tumorigenesis in each group of the transplanted tumor model, the tumor sizes and volumes (tumor volume = (long diameter × short diameter^2^) /2) were calculated. Besides, 2 d after the last administration, a 7% chloral hydrate solution was injected intraperitoneally at a 5 mL/kg dosage. Following the anesthesia process, the tumor masses were stripped, and the volumes and weights were measured. Subsequently, the tumor inhibition rates were determined as follows: Tumor inhibition rate = (average tumor volume of the solution control group - average tumor volume of the administration group) × the average tumor volume of the solution control group^-1^× 100% or (average tumor weight of the solution control group - the average tumor weight of the administration group) × the average tumor weight of the solution control group^-1^×100%. The tumor masses were washed in a normal saline solution. Three nude mice tissues in each group were randomly collected for fixation with 4% paraformaldehyde. Then, after embedding with paraffin, sectioning, de-waxing, rehydration, HE-staining, and dehydration sealing, the histopathological changes were observed under light microscopy.

### Western blot

Two tumor blocks of the nude mice were randomly selected from each group and then cut into pieces. The total protein of the fresh tissue was extracted following the kit instructions. The protein concentrations were measured using a BCA method. The samples' amounts were uniformly adjusted to 30 μg, and subjected to 10% SDS-PAGE to separate protein; then the blot was blocked with 5% skimmed milk powder overnight at 4 °C. Following the washing, the membrane was incubated with MMP2, MMP9, Bax, Bcl-2, and caspase-3 primary antibody (1:1000) and secondary antibody (1:1000), respectively. The chemiluminescent immunoassay was used to develop the blot. The blot image was acquired by an imaging system.

### RNA-Seq analysis

Two tumor blocks of the nude mice were randomly selected from the recombinant Hespintor treatment group and the solvent control group. These were immediately placed into liquid nitrogen, and then quickly transferred to -80 °C for preservation. The Chongqing Western Biotechnology Co. completed the subsequent RNA extractions and testing from the fresh tissue samples, library construction, and computer sequencing. Then, after the original sequencing sequence was obtained, the sequencing data's quality was first evaluated. Following this, the original data's quality control, reference sequence alignment, and splicing of transcripts were carried out after the original sequencing sequence was determined to have met the standards.

### Screening and co-expression analysis of the differentially expressed gene sets

The screening criteria for the differentially expressed genes were as follows:│log FC│≥2, *P*<0.05, adj. *P*<0.05. The differentially expressed genes obtained through the screening process were submitted to the Metascape database (http://metascape.org) for enrichment analysis of the GO molecular function, GO biological process, GO cellular component, and KEGG pathway. The enrichment standard was Min Overlap≥2, *P*-Value Cutoff≤0.05, and the rest was the default value 6. The screened differential expression genes were submitted to the String online tool (http://string-db.org) to build a visualized interaction network. The parameters were set to the mean of the network edges: Confidence; Active interaction sources: Textmining, Experiments, Databases; minimum required interaction score≥0.7 (full score: 1); hide disconnected nodes in the network; select the default value for the remainder.

### Identification of the target genes and construction of a regulatory network

The gene set with the interaction relationship was imported into the Cytoscape software to build a visualized network. First, the core genes that constituted the network's stable structure were screened out using an MCODE plug-in. The parameters were set as Degree Cutoff≥3, K-Core≥4, and the remainder was set as default values. Then, the topological characteristics of the network and each node were calculated using a Centiscape plug-in. It was specified that the gene corresponding to the node with a degree value ≥ mean + SD was the key (hub) gene, and the gene corresponding to the node with a betweenness value ≥ mean + SD was the bottleneck gene. Then, the main path names were summarized and obtained by the annotation by taking the intersection of the three genes as the target gene. The mutual relationship text was established and imported into the Cytoscape software to build a visual regulatory network.

### Statistical analysis

The western blot bands were analyzed using Image J software. The targeted proteins' relative expressions were calculated as follows: The specified group intensity of the targeted protein bands/the specified group intensity of the house-keeping protein bands. The data were analyzed with a one-way analysis of variance followed by the student's *t-*test. All of the data were presented as means and standard deviations.

## Results

### Inducible expressions, purification, and activity detections of the recombinant Hespintor

**Figure 1:**
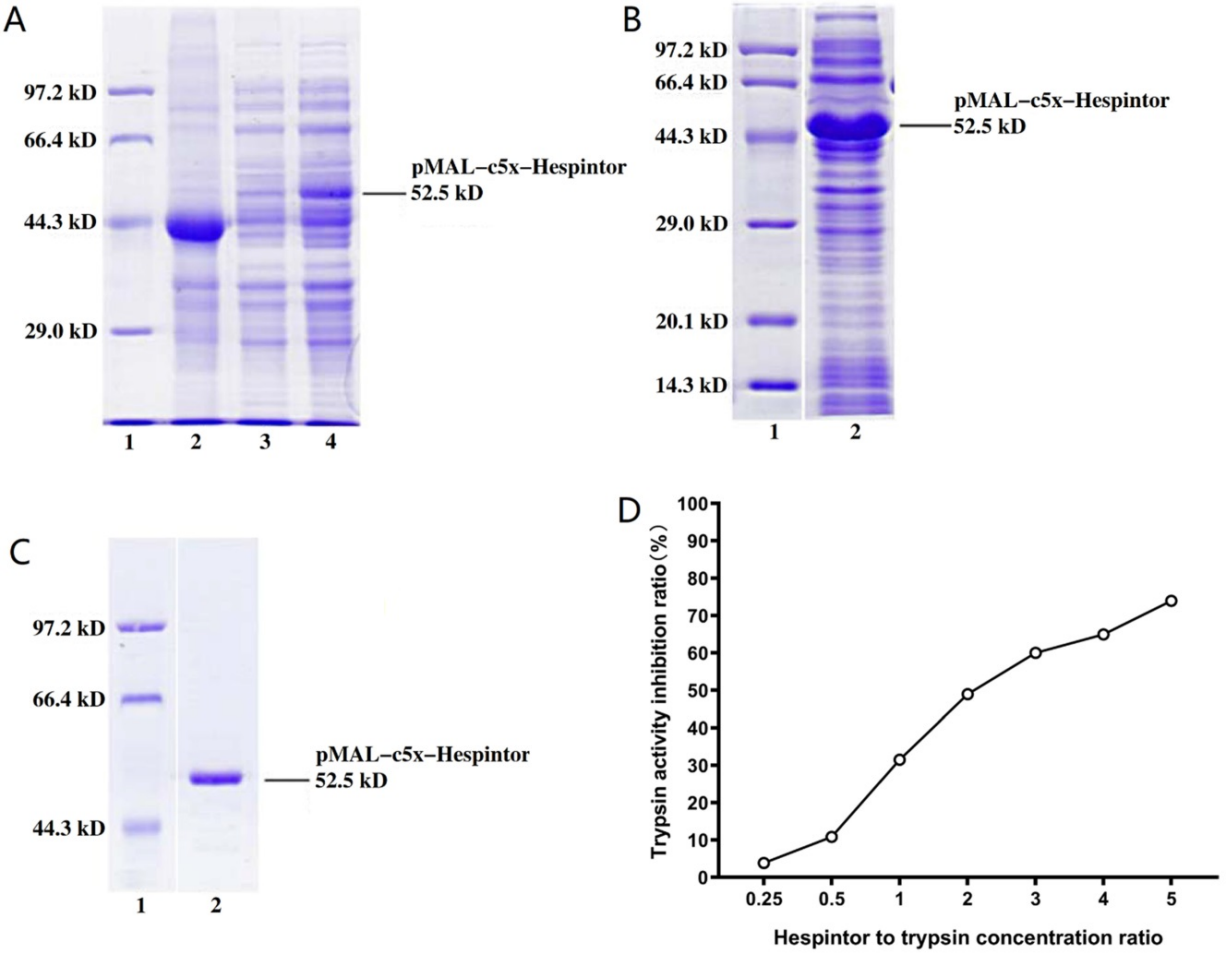
** Induced expression, purification, and activity identification of the recombinant Hespintor.** (**A**) Induced expressions of the recombinant Hespintor test (0.8 mm IPTG; 37 °C for 4 h); 1: Easysee Western marker; 2: Induced expression of BL21(DE3)/pMAL-c5x; 3. Uninduced expression of BL21(DE3)/pMAL-c5x-Hespintor; 4. Induced expression of BL21(DE3)/pMAL-c5x-Hespintor; (**B**) Formal induced expressions of the recombinant Hespintor (0.2 mm IPTG; 18 °C for 16 h); (**C**) Purified recombinant Hespintor; (**D**) Inhibition of the purified recombinant Hespintor on the hydrolysis activities of trypsin. Note: When the mass ratios between the recombinant Hespintor and the trypsin were 1:4, 1:2, 1:1, 2:1, 3:1, 4:1, and 5:1, the respective inhibition rates of the Trypsin activities were 3.84, 10.87, 31.51, 49.00, 60.00, 64.98, and 73.99%, respectively.

Following the induced expression test, the BL21(DE3)/pMAL-c5x-Hespintor could express the recombinant protein (**Fig. 1A**). Following the formal induction and expression processes, the bacteria were collected by centrifugation, and 1/10 volume of lysozyme buffer and lysozyme were added. Then, the bacteria were resuspended for precipitation purposes. The bacteria were broken by ultrasound on ice until the bacteria solution was observed to be clear. The recombinant protein could exist in the lysate supernatant in a soluble protein (**Fig. 1B**). The supernatant was purified using a Ni-NTA affinity chromatography column. The renaturation process of the recombinant protein was completed on a nickel column. Following the one-step elution, the recombinant protein solution was desalted by a semi-permeable membrane and analyzed by SDS-PAGE to obtain a single band (**Fig. 1C**). It is known that trypsin can hydrolyze the low molecular substrate BAPNA to release yellow p-nitroaniline, which has a maximum absorption value at 405 nm. Therefore, if a Trypsin inhibitor is added to a reaction system, then the trypsin's hydrolytic activities should be inhibited, and the absorption value of A405 nm would be decreased. This study's results revealed that 10μg of recombinant protein and 10μg of trypsin had effectively inhibited 31.51% of the trypsin activity, as shown in **Fig. 1D**.

### Inhibitory effects of the recombinant Hespintoron MHCC97-H cells in-vivo

**Figure 2:**
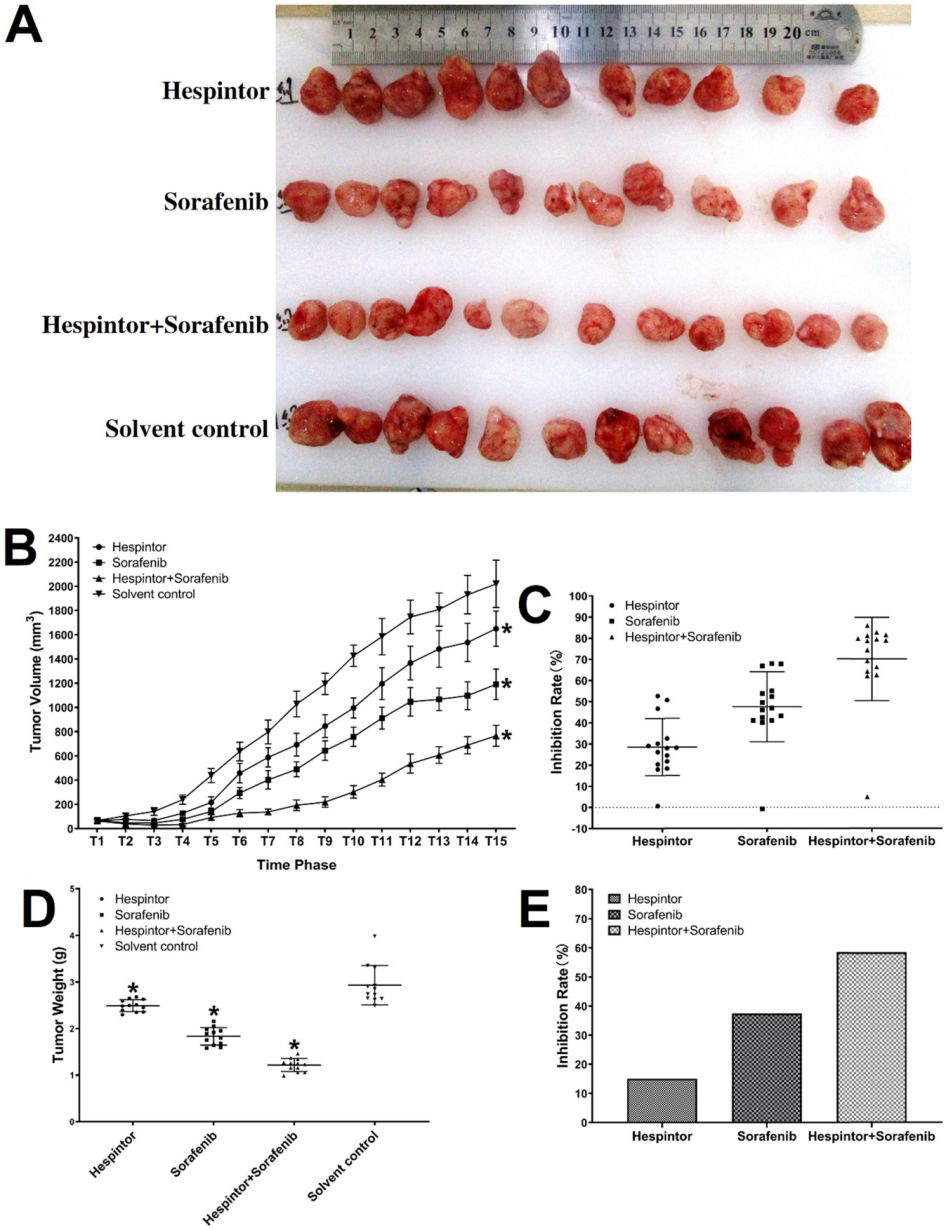
** Inhibitions of the subcutaneous growth of MHCC97-H cells in nude mice by recombinant Hespintor combined with Sorafenib.** MHCC97-H cells were injected into nude mice to form subcutaneous tumors. The mice received recombinant Hespintor, Sorafenib, or recombinant Hespintor combined with Sorafenib treatments via tail vein injections. The results are shown as (**A**) Images of tumors; (**B**) Volumes of tumors (*P<0.05 versus solvent control after T6 phase); (**C**) Inhibition rates from volumes of tumors; (**D**) Weights of tumors (*P<0.05 versus solvent control); (**E**) Inhibition rates from weights of tumors. Note: In the figure, the inhibition rates of the tumor weights are 15.04, 37.48, and 58.55%, respectively.

**Figure 3:**
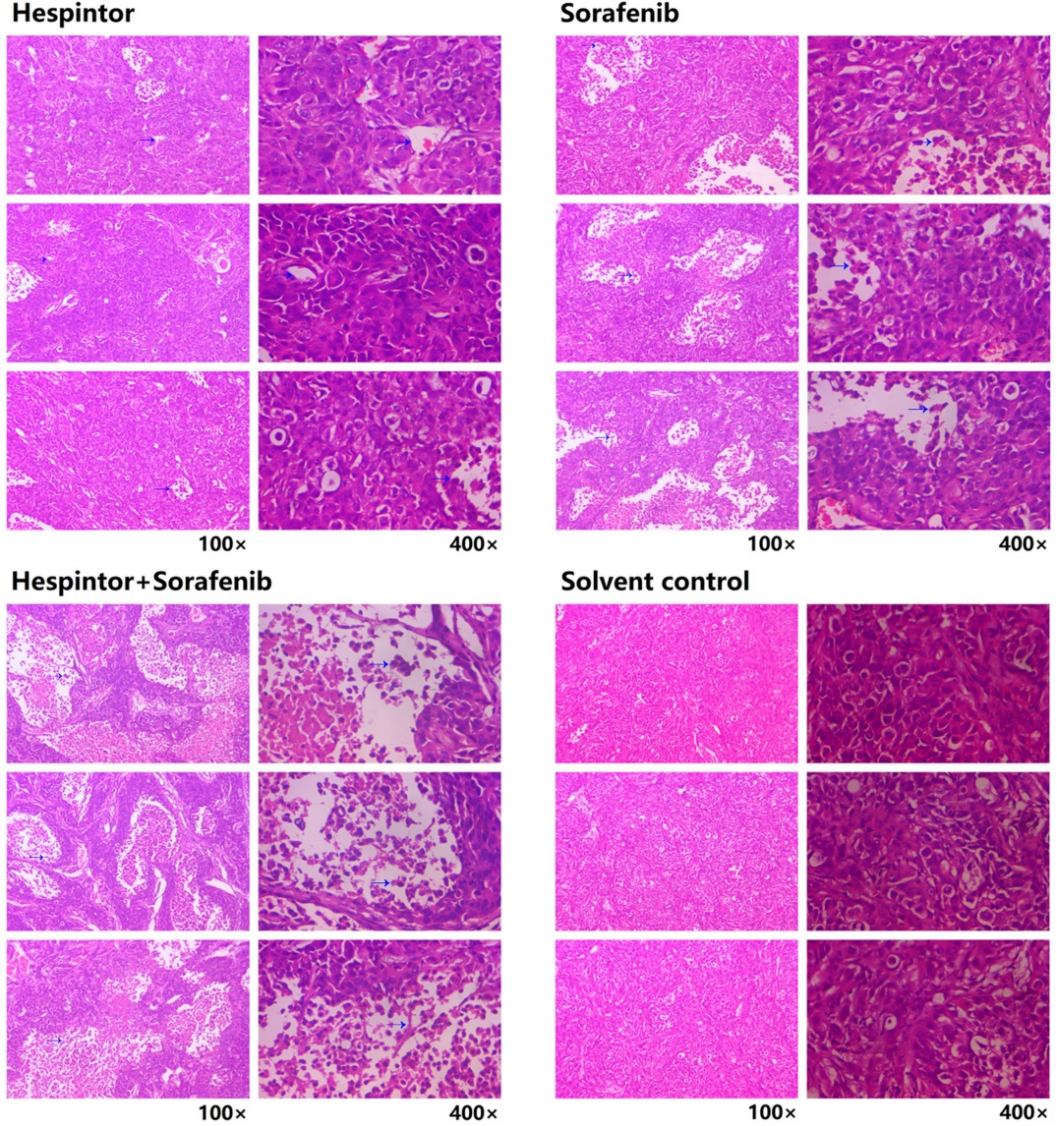
** Histopathological changes in the tumor tissue observed by HE-staining.** The blue arrow points to the necrotic area.

The transplanted tumor model was obtained by subcutaneous injections of MHCC97-H hepatoma cells into nude mice. Three days after the tumor-bearing, solutions containing 20μg/kg of Hespintor and 2mg/kg of Sorafenib were injected the nude mice's tail veins either at the same time or separately. These treatments were conducted continuously for 4 weeks. The tumor volumes were measured every other day. Finally, the tumors were stripped and weighed to calculate the tumor inhibition rates. As shown in **Fig. 2**, both the recombinant Hespintor treatment and Sorafenib treatment nude mice groups had displayed significantly inhibited subcutaneous growth of the MHCC97-H hepatoma cells compared with the solution control group. Meanwhile, the combined treatment group had even more significant antitumor effects. During the dissections of the nude mice's celiac desquamations, it was observed that three of the livers from the solution control group exhibited visible metastasis. Besides, the livers were found to have multiple metastasis nodules of different sizes and with diffuse distributions. The HE-staining of the tumor tissues revealed that when compared with the solvent control group, the cells of the group were densely arranged, and the necrosis areas appeared in the tumor tissue centers following the different treatments of Hespintor or Sorafenib. However, it was observed that the necrosis areas in the Hespintor treatment group were smaller than those in the Sorafenib treatment group. Meanwhile, the necrosis areas in the combined treatment group's tumor tissue centers were significantly enlarged and increased obviously (**Fig. 3**). These results indicated that the treatments had effectively inhibited the excessive proliferation of liver tumor cells.

### Examination of the expressions of the MMP2, MMP9, Bax, Bcl-2, and caspase-3 in the tumor tissue

**Figure 4:**
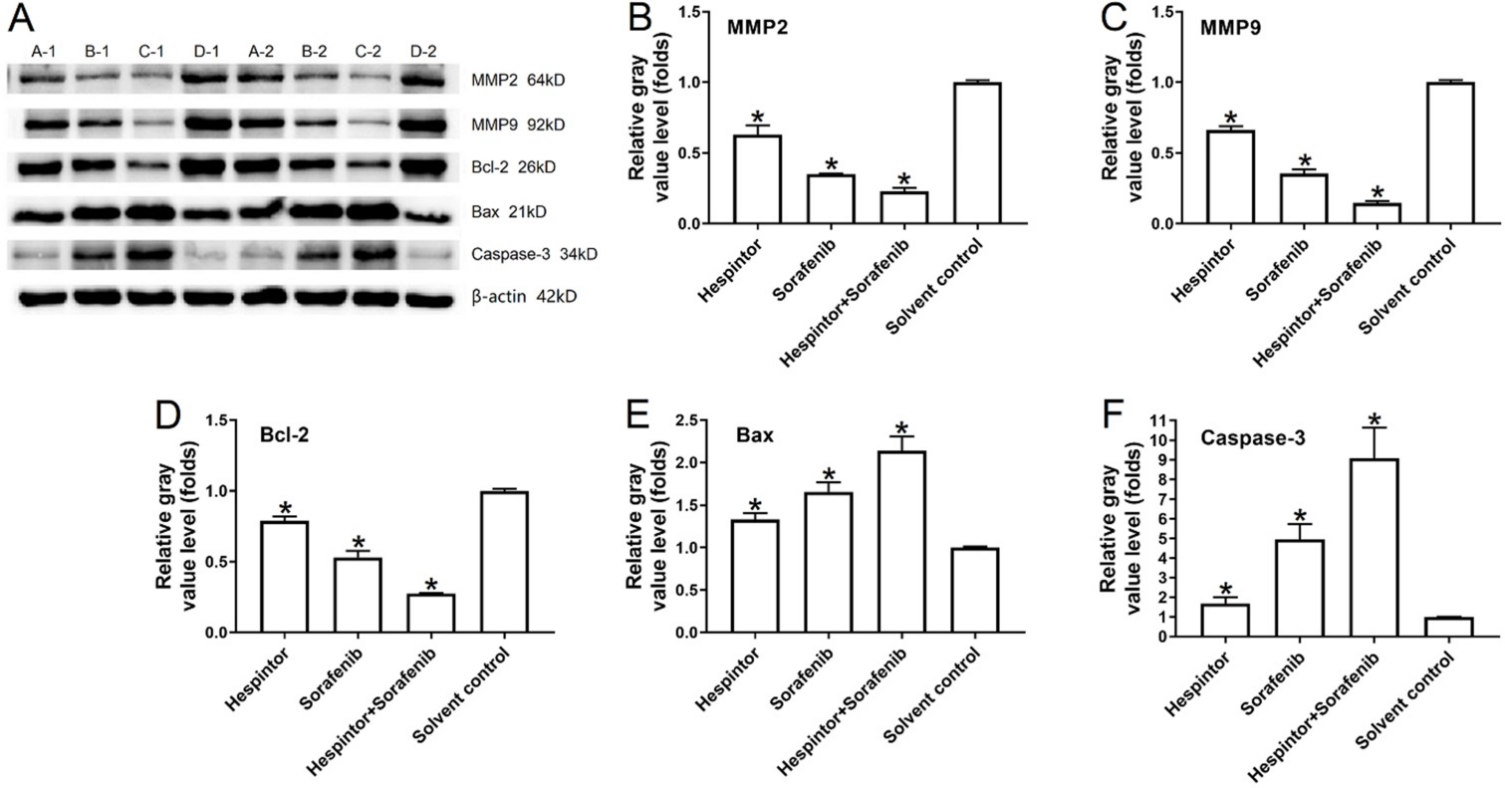
** Recombinant Hespintor combined with Sorafenib regulations of the expressions of MMP2, MMP9, Bcl-2, Bax, and caspase-3 in the tumor tissues of nude mice.** Note: In the figure, the amounts of MMP2, MMP9, Bcl-2, Bax, and caspase-3 were examined using a western blot Method; the results are shown as (**A**) Images; and (**B to F**) Quantitative analysis results, *P<0.05 versus solvent control. A-1 and A-2: recombinant Hespintor treatment group, B-1 and B-2: Sorafenib treatment group, C-1 and C-2: combined administration group, D-1 and D-2: solvent control group.

This study's western blot results showed that the recombinant Hespintor group and the Sorafenib group displayed significantly inhibited expressions of MMP2 and MMP9 in the nude mice's tumor tissues. Simultaneously, the groups also displayed inhibited expressions of Bcl-2 and promoted expressions of the apoptosis-related factors Bax and caspase-3. The combined treatment group's effects were still observed to be significantly better than those of the single-drug treatment groups, which were found to be consistent with the tumor inhibition results in-vivo (**Fig. 4**).

### Quality evaluations of the RNA-Seq sequencing

The cDNA libraries of the recombinant Hespintor treatment group and the solution control group were constructed for the transcriptome sequencing in this study. The obtained original data were filtered, checked for sequencing error rates, and checked for GC content distributions to remove the low-quality data. Then, the effective sequencing data (clean reads) were compared to the human reference genome. The 91230446 and 98123016 effective sequencing data (clean reads) were obtained in the recombinant Hespintor treatment group and the solution control group. The quality values of Q30 were 92.9% and 94.07%, respectively, and the GC content levels were 49.23% and 51.39%, respectively. The transplanted tumors' comparison rate between the recombinant Hespintor treatment group and the reference gene group was 85.9%. The comparison rate of transplanted tumors between the solvent control group and the reference gene group was 82.85%. The ratios of the multiple mapped reads on the reference sequence were 3.79% and 4.52%, respectively. The results mentioned above indicated that the sequencing quality was high, and the results were considered reliable, which successfully met the requirements of follow-up analysis processes. The comparison results were spliced into transcripts, and the levels of the mRNA expressions were quantified by fragments per kilobase of exon per million reads mapped (FPKM). On this basis, differential expression genes or transcripts were screened. The default screening criteria were: │log FC│≥1, *P*<0.05, adj. *P*<0.05. In this study, a total of 1,466 differential expression genes and 21,749 transcripts were obtained.

### Screening and co-expression analysis of the differentially expressed genes

To improve the significance analysis's accuracy level, 979 differentially expressed genes were screened according to the self-defined screening conditions. These included 539 up-regulated genes and 440 down-regulated genes.

**Figure 5:**
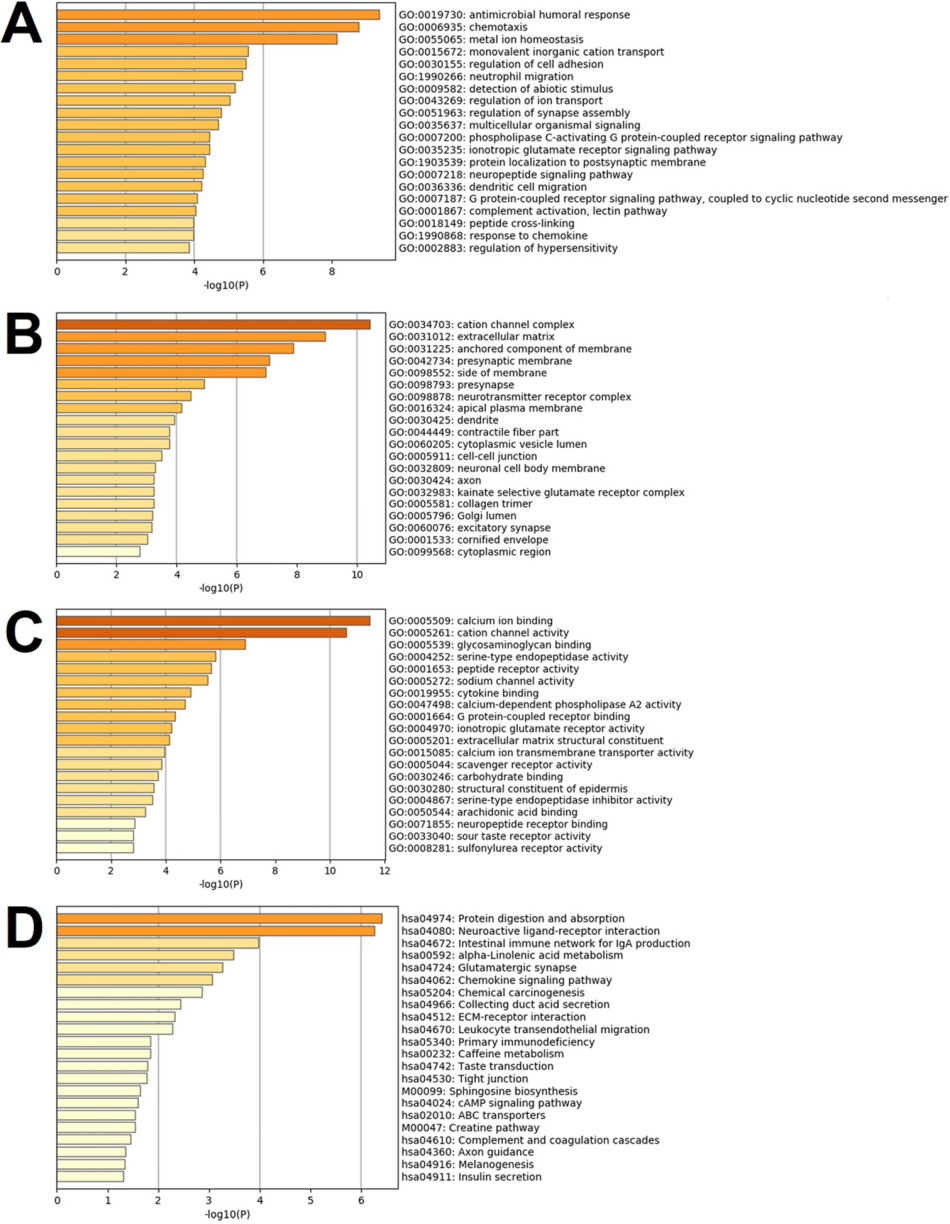
** Results of the gene set enrichment analysis of the differentially expressed genes.** Note: In the figure, the results are shown as (**A**) Images of top 20 of the biological process term of the GO ranked by *p*-value and number of genes; (**B**) Images of the top 20 of the cellular component term of the GO ranked by *p*-value and number of genes; (**C**) Images of the top 20 of the molecular function term of the GO ranked by *p*-value and number of genes; (**D**) All of the KEGG pathway terms ranked by *p*-value and number of genes.

Differentially expressed genes are known to be significantly enriched in the inflammatory responses, immune regulations, cell behaviors, ion transport, neural regulations, G protein-coupled receptor signal pathways, and other functions of the biological processes of the GO. These have adapted to the many enrichment results of the GO's molecular functions, such as the interaction and activity regulations. Multiple ion channels, enzymes, enzyme inhibitors, and receptor activities are regulated by binding ions, polysaccharides, receptors, fatty acids, and immune factors, thereby affecting the above-mentioned biological processes of the GO. These functions are reflected in their close relationships to the functions of the ionic channels and receptor complexes, nerve membrane, nerve endomembrane, extracellular matrixes, endoplasmic cavities, collagen, etc. **Fig. 5A to 5C** detail the enrichment analysis results of biological processes of the GO, cellular components of the GO, and molecular functions of the GO.

**Figure 6:**
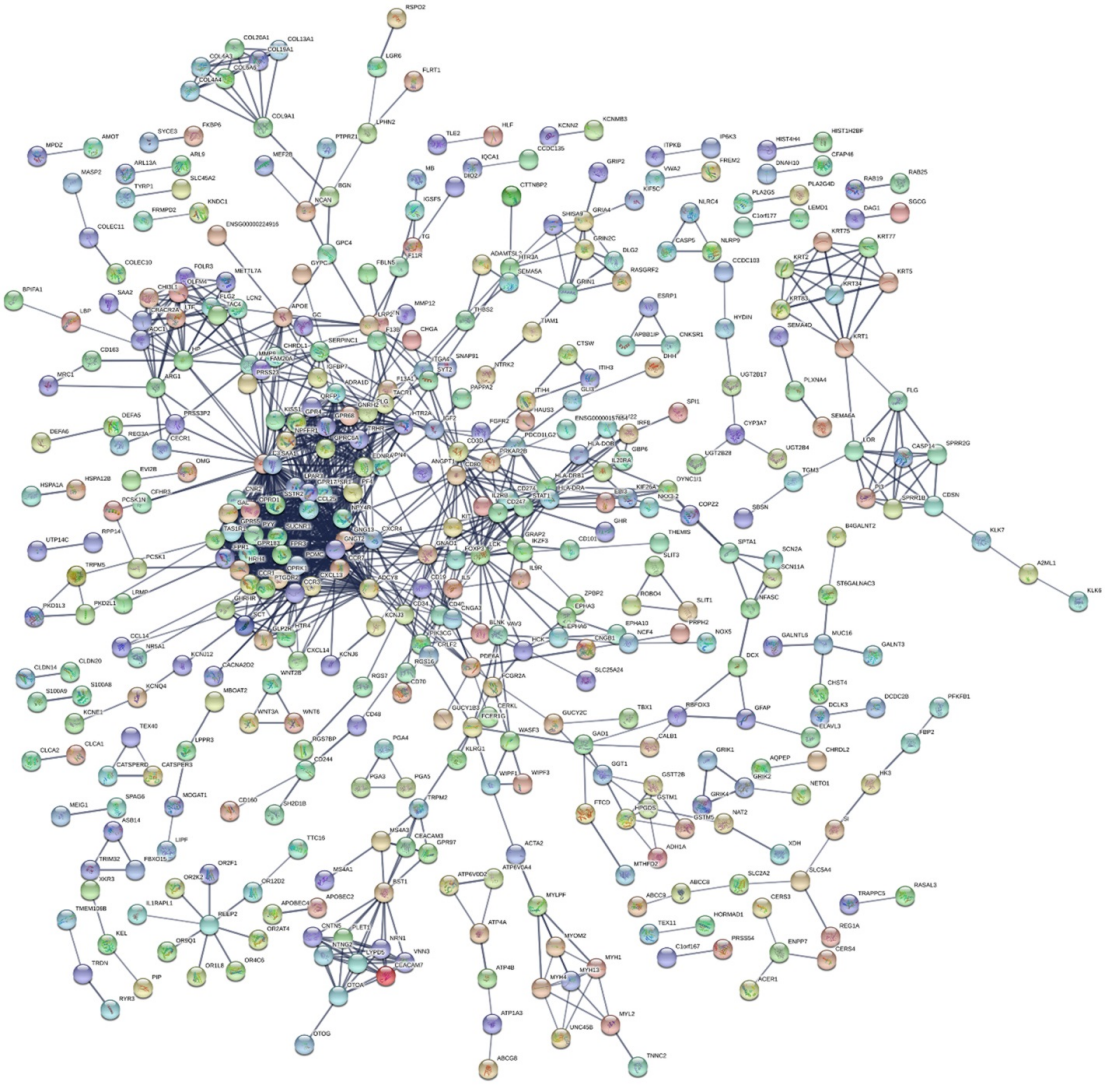
Protein-protein interaction network analysis results.

This study's KEGG pathway analysis results revealed that the differentially expressed genes were significantly affected by the protein digestion and absorption processes; neuroactive ligand-receptor interactions; IgA (intelligent immune network for IgA production); chemokine signaling pathways; ECM (extracellular matrix) receptor interactions; tight junctions; cAMP signaling pathways; complement and coagulation cascades; insulin secretion; and multiple metabolic pathways. The enrichment analysis results of the KEGG pathways are shown in **Fig. 5D**. In the present study, after uploading the differentially expressed genes to the online string tool, and then screening according to the set parameter standard, 235 genes with repetitive and non-interaction isolation were removed, and a comprehensive interaction network consisting of 953 relationship pairs of the remaining 744 node proteins was constructed (**Fig. 6**).

### Identification of the target gene of Hespintorand constructing its regulatory network

**Figure 7:**
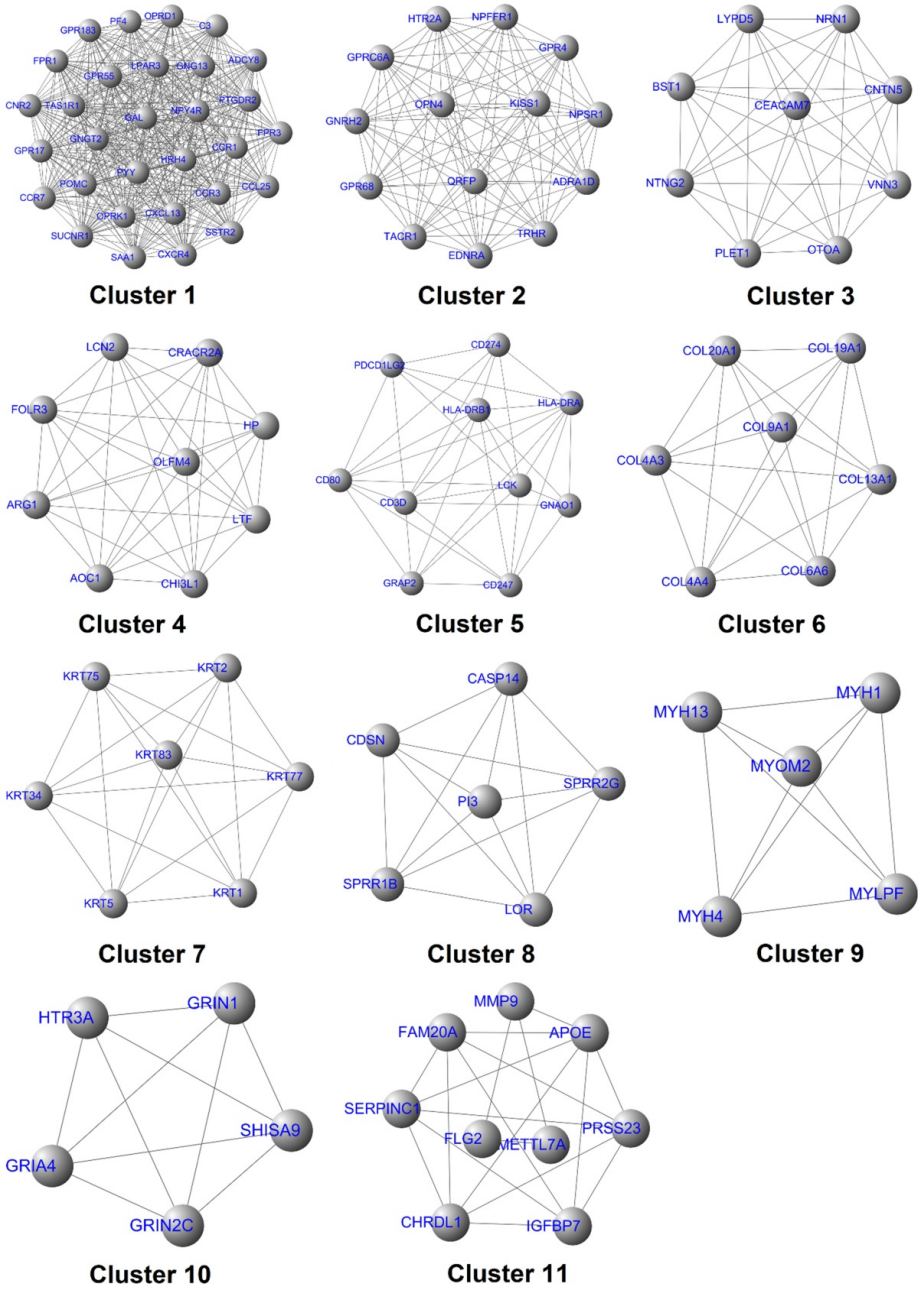
The interaction diagram of the core genes.

**Figure 8:**
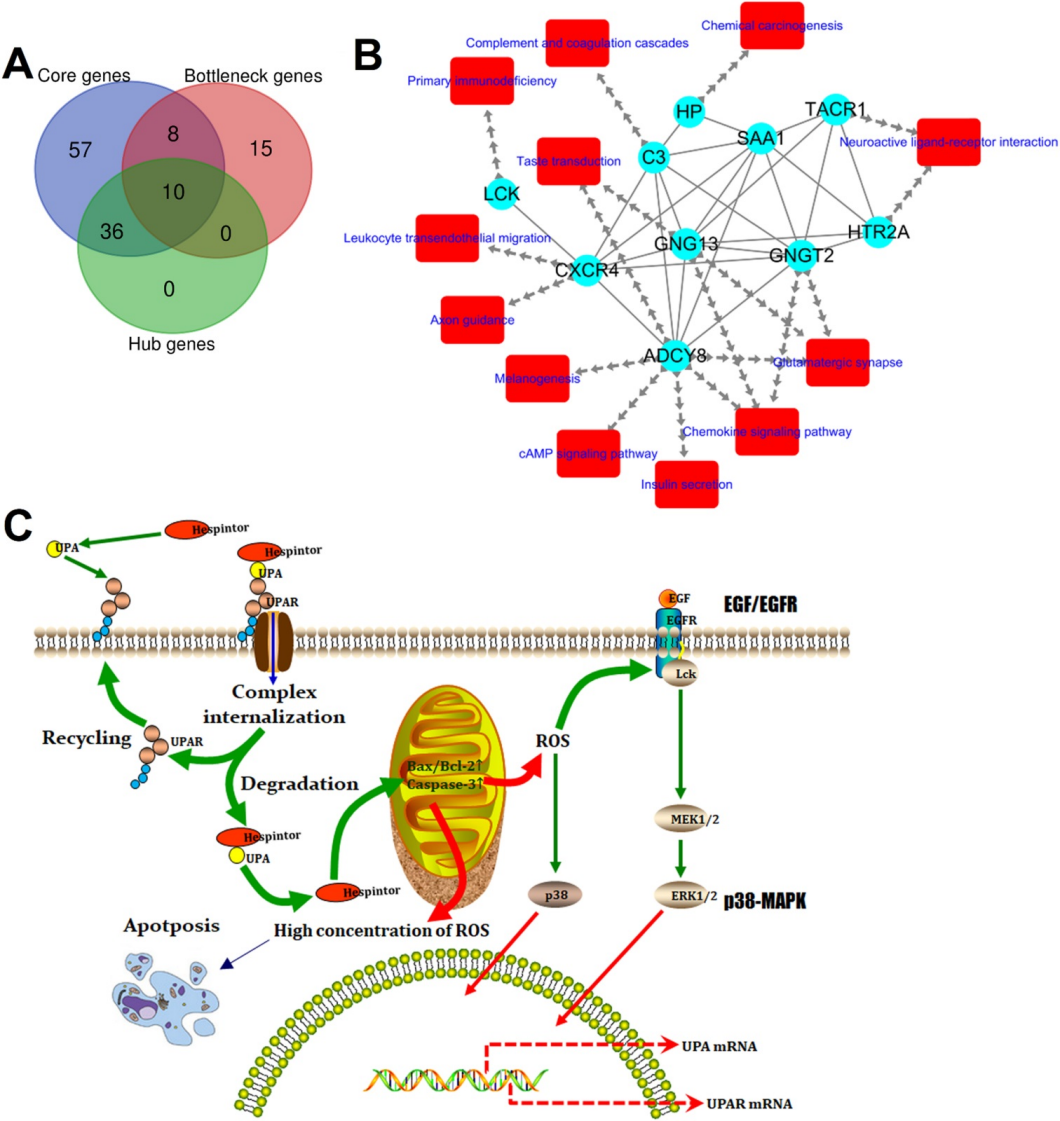
** Identification of the target genes and construction of its dynamic regulatory network.** (**A**) Venn analysis results of the core genes, hub genes, and bottleneck genes; (**B**) Regulatory network of the target genes, in which the blue ellipse node and red round rectangle node represent the target gene and pathway, respectively, and the separate arrow line and solid line represent the target gene-pathway and target gene-target gene, respectively; (**C**) Model of the presumed mechanism of Hespintor inducing cell apoptosis via mitochondrial apoptosis pathway. Hespintor induced ROS via the mitochondrial apoptosis pathway. The medium-low concentration of ROS enhanced Lck expression and phosphorylation (p56*^lck^*), the latter of which activated p38-MAPK and EGF/EGFR pathway to promote uPA and uPAR expression; High concentration of ROS induced cell apoptosis.

The above-mentioned protein interaction network was imported into Cytoscape software for this study's visualization process. The “MCODE” plug-in of the software was used for the K-core analysis of the network. Then, according to the set parameter standards, 11 stable core gene sets (**Fig. 7**) of the network were obtained after the screening, which included 111 core genes. The network's topological characteristics and each node were calculated using the “CentiScape” plug-in of the Cytoscape software. The degree represented the total number of the connected edges of the nodes. The maximum value was 55, and the minimum value was determined to be 1, with an observed average value of 6.08. Besides, after screening, according to the set parameter standards, 46 key genes were yielded. The betweenness indicated the proportion of the number of shortest paths passing through this node in the network. The maximum value was determined to be 15,753.95; the minimum value was 0, and the average value was 631.68. Once again, after screening according to the set parameter standards, 33 bottleneck genes were obtained. Simultaneously, those core genes, the key genes, and the bottleneck genes were crucial to the network were identified. The Hespintor target genes (10 intotal) were successfully determined, as detailed in **Fig. 8A.** These genes included C-X-C motif chemokine receptor 4 (CXCR4), 5-hydroxytryptamine receptor 2A (HTR2A), G protein subunit gamma transducin 2 (GNGT2), LCK proto-oncogene, Src family tyrosine kinase (LCK), adenylate cyclase 8 (ADCY8), serum amyloid A1 (SAA1), tachykinin receptor 1 (TACR1), complement C3 (C3), G protein subunit gamma 13 (GNG13) and haptoglobin (HP). Among these genes, the CXCR4, LCK, and ADCY8 were up-regulated genes, and the remainder was down-regulated genes, as shown in **Table 1.**

All of the interaction relationships between the target genes, along with the main pathways annotated by them, were imported into the Cytoscape software to construct a visual regulatory network of the target mRNA pathway (**Fig. 8B**), which consisted of 22 nodes (10 target genes and 12 pathways) and 45 relationship pairs, respectively.

## Discussion

The hydrolysis of the tumor cells' extracellular matrix is an important step in tumor invasion and metastasis. It has been observed that after urokinase-type plasminogen activator (uPA) secreted by the tumor cells, specifically binds to its receptor uPAR, the plasminogen on the cell surface is transformed into plasmin 7. Previous investigations have revealed that plasmin cannot only directly degrade the matrix components of fibronectin, laminin, and proteoglycan, but also activate MMPs to induce focal proteolysis of ECM and basement membrane (BM), as well as to promote the migration of tumor cells, immune cells, and vascular endothelial cells 8. Therefore, by inhibiting the serine protease activities of the uPA, serpins can indirectly inhibit the activities of the MMPs, thereby preventing the invasion or (and) migration of tumor cells 9. As a serine protease inhibitor, Hespintor inhibits the direct cleavage and activation of MMP by interacting with uPA, and its expression in non-tumor samples is significantly higher than that in hepatocellular carcinoma samples, especially in advanced hepatocellular carcinoma samples 5. Except for our group's previous work, it has been reported that Hespintor can promote ovarian cancer metastasis after down-regulated expression of siRNA 10, which shows that Hespintor has strong antitumor activity. Based on the previous studies, the human hepatoma cell line MHCC97-H with high metastatic potential was selected to mimic advanced hepatocarcinoma's highly invasive characteristics 11. In previous research, this research group successfully obtained the high-efficient expression of recombinant Hespintor and achieved the protein purification and refolding step of the group, including body weight 4. In this study, the Hespintor coding gene sequence codon was optimized, but the pMAL-c5x prokaryotic expression vector was also used to realize the target gene's fusion expression maltose-binding protein (MBP). Furthermore, the fusion protein's soluble expression was realized by the malE signal sequence guiding the fusion protein through the cell membrane.

As an inhibitor of a variety of tyrosine kinases, Sorafenib can act on tumor cells and tumor blood vessels simultaneously, thereby displaying dual antitumor effects 12. However, since Sorafenib can block multiple kinase signaling pathways, thereby leading to cancer cell proliferation and drug resistance, and is known to have strong side effects, it has been necessary to find ways to reduce the side effects of Sorafenib and improve its the efficacy 13. In clinical treatments, it has been found that when Sorafenib is combined with chemotherapy medications or targeted drugs, more obvious therapeutic effects and less toxic side effects have been observed 14.

This study showed that recombinant Hespintor injections had displayed good antitumor effects on the subcutaneous growth of MHCC97-H cells. In particular, the combination therapy effects were found to be significantly better than those of the single therapy methods. In terms of the tumor inhibition rates, histopathology, and inhibition of MMP2 and MMP9, it was observed that although the Hespintor was not as effective as Sorafenib, two factors should be considered. On the one hand, the dosages of Hespintor were low, and its therapeutic effects were not ideal. However, on the other hand, the Sorafenib had been administered according to the conventional dosages. Due to its toxic side effects, it had affected not only the growth of transplanted tumors in the nude mice but also the growth of transplanted tumors in the nude mice affected the weight gain rates. The nude mice's physical conditions in the Sorafenib treatment group were significantly lower than those in the Hespintor treatment group. It has been determined that the majority of the current antitumor drugs can induce tumor cell apoptosis and display antitumor effects. The initiation and transduction of apoptosis *in-vivo* are activated and precisely regulated by a variety of apoptotic signals. Apoptosis mainly includes the death receptor pathway, mitochondrion pathway, and endoplasmic reticulum pathway, but they can induce a caspase cascade reaction 15. In the mitochondria apoptosis pathways, the expression ratios of pro-apoptotic Bax and anti-apoptotic Bcl-2 play important roles in apoptosis. The Bcl-2 family regulation on apoptosis is known to be related to the activation of the caspase family, and caspase activation is the key link of the apoptosis. Bax is distributed in the mitochondrial matrix, which can promote cytochrome C's release by altering the permeability of the mitochondrial membrane, activating the caspase-3 via caspase pathways, and subsequent cascade reactions, which subsequently promote cell apoptosis 16. The results achieved in this study showed that the examined drug combination could significantly up-regulate the Bax/Bcl-2 ratios and induce the expression of caspase-3, thereby activating the expressions of the pro-apoptotic factor Bax and caspase-3, and inhibiting the expressions of the anti-apoptotic factor Bcl-2. Therefore, it was speculated that the mitochondrial apoptosis pathways might be one of the mechanisms of Hespintor induced apoptosis.

It has proven helpful in determining the relationships between “diseases and genes” using network module division methods. At present, the concept of “network structure entropy” is the best way to determine a network module. The smaller the network module's entropy values are, the higher the similarity between nodes will be, and the more stable and reliable the module will be. Meanwhile, after the network modules are divided using MCODE cluster methods, the entropy values will be the minimum values 17. In this study, the significant differential expression target genes of Hespintor acting tumor tissue were screened out based on RNA-Seq. As a result, the interactions between molecules and possible regulatory pathways were more clearly understood using the visual regulatory network. The main focus of this study's investigations was on the cell migrations, inflammatory responses, cell behaviors, neural regulations, metabolic regulations, cell communications, and other functions, which were determined to be closely related to the occurrence and development of tumors. The serpins' acting mechanism in the tumors was found to be very complex, having displayed contradictory expressions of inhibition and promotion. Also, it was not limited to one action against proteolysis. Another reason for the serpins' complex mechanism was that the ultimate substrate MMPs family was very large, with many different functions. In addition to its interactions with the upstream and downstream targets, the interactions between members of the MMPs family were also observed to affect the tumor cells 18. Therefore, given those factors, the serpins' mechanism in the malignant tumors will require further in-depth study and specific analyses in the future.

CXCR4 is one of the most important chemokine receptors since it promotes tumor cell metastasis by forming the CXCL12/CXCR4 biological axis 19. The Src family tyrosine kinase LCK is mainly involved in regulating the T cell development and homeostasis and has been used in CAR-T immunocytes therapy. Since it has actively regulated proliferation, survival, and other tumor cells' functions, it has become a new drug target for tumor and neurological disease treatments 20. In the present study, it was found that the expressions of CXCR4 and LCK in the tumor tissue of the Hespintor treatment group were significantly higher than those of the solvent control group, which appeared to be contrary to the general theory. However, this was not the case. The regulation of reactive oxygen species (ROS) on the cancer cell signaling pathway has two sides.

On the one hand, a low concentration of ROS can activate the signal pathway related to proliferation, survival, angiogenesis, and metastasis, and promote the occurrence, development, and metastasis of cancer. On the other hand, a high ROS concentration can induce apoptosis, promote cell aging, and inhibit cell cycle. It was determined that the Hespintor induced apoptosis led to the release of O^2-^ from the mitochondria to the cytoplasm. The ROS were spontaneously generated or catalyzed by the superoxide dismutase (SOD). Then, the ROS induced LCK expressions and phosphorylation (p56*^Lck^*), p56*^Lck^* activated p38 MAPK, and EGF/EGFR pathways promoted the expressions of uPA and uPAR, respectively (**Fig. 8C**) 21, 22. It has been previously reported that 3'-UTR in uPAR and CXCR4 contains the common miRNAs response elements. In other words, up-regulated expressions of uPAR will tend to lead to up-regulated expressions of CXCR4^23^, indicating that the Hespintor could inhibit the invasion and migration of tumor cells and induce apoptosis only after reaching a certain drug concentration. Otherwise, the insufficient concentration of Hespintor would be easy to induce ROS generation and promote the expressions of uPA by relying on the mediating role of ROS, which could explain the contradictory expressions of serpins in the tumors examined in this study to a certain extent. Besides, the methylation of the ADCY8 gene promoter, which is related to the cytological classification, is considered to be a biomarker of cervical cancer. The methylation levels of the ADCY8 gene promoter are consistent with the malignancy degrees of tumors. Following treatments, the up-regulated expressions of the ADCY8 gene indicate decreases in the degrees of malignancy 24. The seven down-regulated target genes are considered potential biomarkers and/or therapeutic targets of hepatocellular carcinoma or other tumors. The majority of these genes are directly involved in signal transduction, which also indicates a good prognosis. According to the function and mechanism of action, these can be divided into three categories: (1) HTR2A as a neurotransmitter receptor, and TACR1 as a neuropeptide receptor, both of which are related to cell proliferation 25, 26; (2) GNGT2 and GNG13 are G proteins coupled receptor components, where the former promotes cell proliferation, and the latter mediates cell responses 27, 28; (3) SAA1, C3, and HP are regulatory components related to cell migration, among which the SAA1 induces IL8/CXCL8 promoter activity; C3 enhances the expressions of p-JAK2/p-STAT3 in gastric cancer cells, and HP is involved with antioxidant activities 29-31.

In summary, combined with our previous study 5, the antitumor effects of Hespintor were preliminarily proved in a concentration-dependent manner. Of course, the results still need to be verified in various transplanted tumors in nude mice. Following the interactions between Hespintor and uPA, Hespintor enters cells through uPAR-mediated internalization. The mechanism of action of the Hespintor involved multiple signaling pathways. For example, there was more than an antitumor mechanism of extracellular inhibition of uPA activities involved in the actions. Therefore, it is possible that other biological functions of the Hespintor exist, but have not yet been identified.

## Supplementary Material

Supplementary tables.

## Figures and Tables

**Table 1:** Identification result of target genes

Gene class	Gene name
Core genes	*PTGDR2, CXCR4, CCR3, GPR17, C3, OPRK1, SUCNR1, HRH4, OPRD1, POMC, ADCY8, GNG13, CCR1, GNGT2, LPAR3, SSTR2, CCR7, CXCL13, PYY, NPY4R, GPR183, PF4, CCL25, FPR3, CNR2, GPR55, GAL, TAS1R1, SAA1, FPR1, TACR1, NPFFR1, QRFP, KISS1, GPR4, NPSR1, GNRH2, EDNRA, TRHR, ADRA1D, HTR2A, GPR68, OPN4, GPRC6A, NTNG2, CNTN5, PLET1, CEACAM7, VNN3, LYPD5, OTOA, NRN1, BST1, ARG1, HP, OLFM4, LTF, FOLR3, LCN2, CRACR2A, AOC1, CHI3L1, GRAP2, HLA-DRB1, CD80, HLA-DRA, CD274, LCK, PDCD1LG2, CD247, GNAO1, CD3D, COL9A1, COL6A6, COL4A4, COL20A1, COL13A1, COL19A1, COL4A3, KRT75, KRT2, KRT5, KRT1, KRT77, KRT34, KRT83, CDSN, PI3, LOR, SPRR1B, SPRR2G, CASP14, MYH1, MYH13, MYH4, MYLPF, MYOM2, GRIN1, GRIA4, GRIN2C, HTR3A, SHISA9, FLG2, IGFBP7, APOE, PRSS23, METTL7A, MMP9, CHRDL1, FAM20A, SERPINC1*
Hub genes	*GNGT2, GNG13, SAA1, C3, LPAR3, GPR17, CXCR4, ADCY8, POMC, PF4, CCR7, CCR3, TAS1R1, CCR1, CCL25, NPY4R, FPR1, SSTR2, CXCL13, PYY, OPRD1, OPRK1, GPR55, CNR2, GAL, GPR183, PTGDR2, FPR3, HRH4, SUCNR1, TACR1, NPSR1, LCK, HTR2A, KISS1, ADRA1D, OPN4, EDNRA, HP, NPFFR1, GNRH2, QRFP, GPRC6A, GPR68, TRHR, GPR4*
Bottleneck genes	*FCER1G, PIK3CG, CXCR4, C3, GNGT2, LCK, GPC4, TRPM2, GNG13, PDE6A, APOE, VAV3, BGN, TACR1, LRP2, SAA1, WIPF1, HTR3A, GNAO1, BST1, GAD1, CD3D, ACTA2, GGT1, MMP9, HP, GUCY2C, MYLPF, STAT1, COL9A1, HTR2A, ADCY8, CD19*
Target genes	*CXCR4, HTR2A, GNGT2, LCK, ADCY8, SAA1, TACR1, C3, GNG13, HP*

## Abbreviations

ADCY8: adenylate cyclase 8
BAPNA: Na-Benzoyl-DL-arginine-4-nitroanilide hydrochloride
BM: basement membrane
C3: complement C3
CXCR4: C-X-C motif chemokine receptor 4
ECM: extracellular matrix
FPKM: fragments per kilobase of exon per million read mapped
GNG13: G protein subunit gamma 13
GNGT2: G protein subunit gamma transducin 2
HP: haptoglobin
HTR2A: 5-hydroxytryptamine receptor 2A
LCK: LCK proto-oncogene, Src family tyrosine kinase
MBP: maltose-binding protein
MMPs: matrix metalloproteinases
ROS: reactive oxygen species
SAA1: serum amyloid A1
Serpins: serine proteinase inhibitors
SPINK13: serine peptidase inhibitor Kazal type 13
SSH: suppression subtractive hybridization
TACR1: tachykinin receptor 1
uPA: urokinase-type plasminogen activator
