# Supplementary material for: The Inhibitory Effects of Recombinant Hespintor Combined with Sorafenib on Transplanted Human Hepatoma in Nude Mice, and Transcriptional Regulation of Hespintor Based on RNA-Seq
Source: J Cancer. 2021 Jan 1;12(2):343–57. doi: 10.7150/jca.50500 (PMC7738984; doi:10.7150/jca.50500)
Supplement: Supplementary file 1 — Supplementary tables. [file jcav12p0343s1.pdf]

Table S1 Volume of tumors (cm^3)

Group / Time	Hespintor	Hespintor+ Sorafenib	Sorafenib	Solvent control
Aug 15, 2019	66.24	67.42	63.58	66.89
Aug 17, 2019	76.30	47.84	39.69	106.22
Aug 19, 2019	67.07	47.06	29.08	141.73
Aug 21, 2019	127.72	76.90	33.65	239.76
Aug 23, 2019	216.31	141.18	92.06	438.53
Aug 25, 2019	459.02	294.73	128.67	637.43
Aug 27, 2019	588.77	403.17	137.78	797.65
Aug 29, 2019	693.08	490.00	193.12	1027.64
Aug 31, 2019	846.09	644.27	219.71	1194.00
Sept 2, 2019	995.53	757.79	303.55	1425.65
Sept 4, 2019	1195.91	911.36	405.26	1584.25
Sept 6, 2019	1366.66	1046.59	536.99	1746.27
Sept 8, 2019	1483.09	1066.74	607.73	1807.98
Sept 10, 2019	1536.51	1096.99	689.16	1931.09
Sept 12, 2019	1649.80	1190.90	766.44	2020.28

Table S2 Weight of tumors (g)

Group / Number	Hespintor	Sorafenib	Hespintor+ Sorafenib	Solvent control
1	2.4977	1.9859	1.0574	2.6531
2	2.5134	1.7541	1.2563	2.7273
3	2.4862	1.6853	1.4634	2.9251
4	2.6317	1.9154	1.0563	2.6243
5	2.6531	1.6425	1.2548	2.6421
6	2.5945	2.1557	0.9865	2.9024
7	2.3518	2.0548	1.2314	3.3561
8	2.3966	1.5819	1.1563	3.3124
9	2.2981	1.9547	1.3677	2.8496
10	2.6821	1.5972	1.3425	3.9842
11	2.4558	1.8923	1.2679	2.4961
12	2.3578	1.7982	1.1576	2.7421

Table S3 Gray ratio analysis of Western blot

Gene \ Number	A1	B1	C1	D1	A2	B2	C2	D2
MMP2	37.19	23.96	16.94	69.86	48.01	25.99	15.31	65.87
MMP9	66.99	40.06	16.53	107.62	72.63	39.12	15.66	104.46
Bcl-2	98.79	74.35	36.44	133.31	108.58	72.64	38.95	130.98
Bax	81.34	108.92	138.11	69.53	86.79	112.56	143.41	58.17
caspase-3	13.22	41.32	75.67	7.56	13.06	40.65	71.98	8.25
β-actin	100.00	108.54	107.60	109.53	107.02	110.78	108.47	99.36

Internal gene correction

Gene \ Number	A1	B1	C1	D1	A2	B2	C2	D2
MMP2	37.19	22.07	15.74	63.78	44.86	23.46	14.11	66.29
MMP9	66.99	36.91	15.36	98.26	67.87	35.31	14.44	105.13
Bcl-2	98.79	68.50	33.87	121.71	101.46	65.57	35.91	131.82
Bax	81.34	100.35	128.36	63.48	81.10	101.61	132.21	58.54
caspase-3	13.22	38.07	70.33	6.90	12.20	36.69	66.36	8.30
β-actin	100.00	100.00	100.00	100.00	100.00	100.00	100.00	100.00

Notes: A-1 and A-2, recombinant Hespintor treatment group; B-1 and B-2, Sorafenib treatment group; C-1 and C-2, combined administration group; D-1 and D-2, solvent control group.
